# Pharmacokinetics and tolerance of repeated oral administration of 5-fluorocytosine in healthy dogs

**DOI:** 10.1186/s12917-021-02927-5

**Published:** 2021-06-21

**Authors:** Jérémy Béguin, Matthias Kohlhauer, Eve Laloy, Frédérique Degorce, Baptiste Moreau, Éric Quéméneur, Philippe Erbs, Bernard Klonjkowski, Christelle Maurey

**Affiliations:** 1 UMR Virologie, INRAE, École Nationale Vétérinaire d’Alfort, ANSES, Université Paris-Est, Maisons-Alfort, France; 2 Department of Internal Medicine, École Nationale Vétérinaire d’Alfort, ANSES, Université Paris-Est, Maisons-Alfort, France; 3 Transgene, Illkirch-Graffenstaden, Strasbourg, France; 4 U955 - IMRB Inserm, École Nationale Vétérinaire d’Alfort, UPEC, F-94700 Maisons-Alfort, France; 5 Pharmacology-Toxicology Unit, École Nationale Vétérinaire d’Alfort, Université Paris-Est, F-94700 Maisons-Alfort, France; 6 Anatomical Pathology Unit, Biopôle Alfort, École Nationale Vétérinaire d’Alfort, Université Paris-Est, F-94700 Maisons-Alfort, France; 7 Laboratoire d’Anatomie Pathologique Vétérinaire du Sud-Ouest, Toulouse, France

**Keywords:** Flucytosine, Fluorouracil, Drug-related side effects, Adverse reactions, Pharmacokinetic, Dog, Targeted chemotherapy, Antineoplastic agents

## Abstract

**Background:**

5-fluorocytosine is a pyrimidine and a fluorinated cytosine analog mainly used as an antifungal agent. It is a precursor of 5-fluorouracil, which possesses anticancer properties. To reduce systemic toxicity of 5-fluorouracil during chemotherapy, 5- fluorocytosine can be used as a targeted anticancer agent. Expression of cytosine deaminase by a viral vector within a tumor allows targeted chemotherapy by converting 5-fluorocytosine into the cytotoxic chemotherapeutic agent 5-fluorouracil. However, little is known about the tolerance of 5-fluorocytosine in dogs after prolonged administration.

**Results:**

In three healthy Beagle dogs receiving 100 mg/kg of 5-fluorocytosine twice daily for 14 days by oral route, non-compartmental pharmacokinetics revealed a terminal elimination half-life of 164.5 ± 22.5 min at day 1 and of 179.2 ± 11.5 min, after 7 days of administration. Clearance was significantly decreased between day 1 and day 7 with 0.386 ± 0.031 and 0.322 ± 0.027 ml/min/kg, respectively. Maximal plasma concentration values were below 100 µg/ml, which is considered within the therapeutic margin for human patients. 5-fluorouracil plasma concentration was below the limit of detection at all time points. The main adverse events consisted of depigmented, ulcerated, exudative, and crusty cutaneous lesions 10 to 13 days after beginning 5-fluorocytosine administration. The lesions were localized to the nasal planum, the lips, the eyelids, and the scrotum. Histological analyses were consistent with a cutaneous lupoid drug reaction. Complete healing was observed 15 to 21 days after cessation of 5-fluorocytosine. No biochemical or hematological adverse events were noticed.

**Conclusions:**

Long term administration of 5-fluorocytosine was associated with cutaneous toxicity in healthy dogs. It suggests that pharmacotherapy should be adjusted to reduce the toxicity of 5-fluorocytosine in targeted chemotherapy.

**Supplementary Information:**

The online version contains supplementary material available at 10.1186/s12917-021-02927-5.

## Background

5-fluorocytosine (5-FC) is a pyrimidine compound and a fluorinated cytosine analog, which has been widely used as a sole or adjuvant antifungal agent. In domestic animals, 5-FC is mainly used at the dosage of 50 to 75 mg/kg PO every eight hours to treat mycoses due to *Cryptococcus neoformans*, *Candida* spp. and filamentous fungi like *Aspergillus* spp. [1–5]. In humans, 5-FC induces gastrointestinal disorders, hepatotoxicity, and bone marrow depression [1, 6–11]. In dogs, gastrointestinal disturbances and bone marrow depression are also described, but the overall toxicity is poorly known. Cutaneous rashes on the nasal planum and the scrotum have also been reported, but only after combination of 5-FC and amphotericin B [2, 3]. The mechanism of 5-FC toxicity is not understood [12]. A hepatic and hematological dose-dependent toxicity is mainly suspected, although not all reports support this theory. Conversion of 5-FC to certain metabolites, like 5-fluorouracil (5-FU), is one of the suspected mechanisms. Patients treated with 5-FC have shown detectable amounts of 5-FU in serum leading to bone marrow depression and gastrointestinal adverse events [13–15].

5-FC can be converted by cytosine deaminase into the cytotoxic chemotherapeutic agent 5-FU. 5-FU is then converted to active metabolites, such as 5-fluorouridine monophosphate (5-FUMP) and 5-fluorodeoxyuridine monophosphate (5-FdUMP), which are incorporated into RNA and DNA, interfering with their synthesis and function [16–18]. Moreover, 5-FdUMP is an inhibitor of thymidylate synthetase, leading to depletion of thymidine 5’-monophosphate and thymidine 5’-triphosphate [19]. The alteration in thymidine and deoxyuridine phosphate pools caused by thymidylate synthetase inhibition affects DNA synthesis and integrity, and RNA synthesis and processing. These mechanisms are all thought to play a role in 5-FU cytotoxicity. Previous reports supported the efficacy of 5-FU in the management of canine mammary neoplasia and for various kinds of carcinoma in veterinary medicine [20–23]. However, in dogs 5-FU toxicity (myelosuppression, gastrointestinal toxicity and neurotoxicity) remains a limiting factor [24].

Systemic chemotherapy has been highly successful in cancer management. However, adverse effects to the patient prevent the aggressive dosing needed to exploit the cytotoxic potential. Recent advances in chemotherapy include the development of targeted anticancer agents to increase efficacy while reducing adverse events. Many modalities such as liposomes, polymeric micelles, quantum dots, gold nanocarriers, carbon nanotubes, and mesoporous silica nanoparticles have been developed [25–29]. Transgene expression by an oncolytic viral vector is one of the promising approaches in targeted chemotherapy. For this purpose, TG6002 a Copenhagen strain vaccinia virus deleted of its thymidine kinase gene (*TK*; *J2R*) and a subunit of its ribonucleotide reductase gene (*RR*; *I4L*) and armed with the suicide gene *FCU1* has been developed. TG6002 replication depends on the expression of cellular TK and RR, which is overexpressed in tumor cells. As a consequence, TG6002 has a strong tumor selectivity leading to intratumoral expression of the *FCU1* gene. The latter encodes a bifunctional chimeric protein, which catalyzes the conversion of non-toxic 5-FC into 5-FU [30].

In a clinical trial, intramuscular administration of TG6002 as a sole treatment or combined with short term oral administration of 5-FC was evaluated and well-tolerated in healthy dogs [31]. However, in antineoplastic treatment, 5-FC will be administrated chronically and likely be associated with a different toxicological profile. No reports have investigated the pharmacokinetics and tolerance of 5-FC as a sole agent after repeated oral administration in healthy dogs. The present study’s objectives are to describe the pharmacokinetics and adverse effects of oral 5-FC administration at the dosage of 100 mg/kg twice daily for 14 days in healthy dogs.

## Results

### Pharmacokinetic profile

**Fig. 1:**
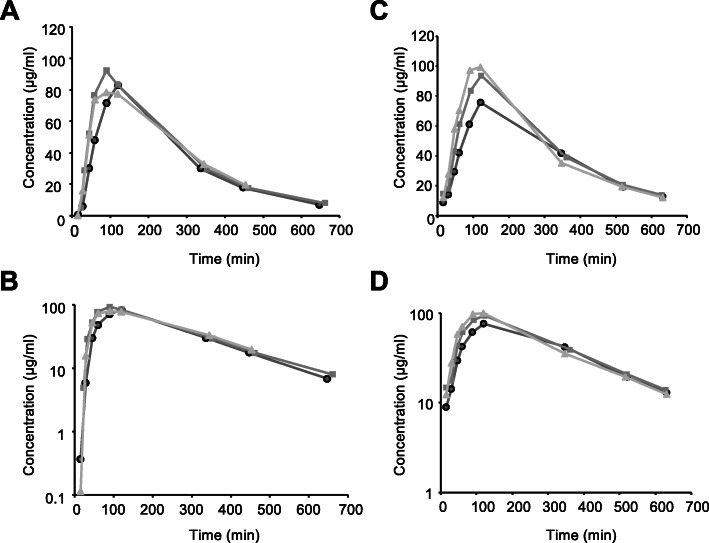
Serum concentration of 5-FC at days 1 and 7 for the three dogs. **A**: Linear plot of individuals plasma concentration-time profile of 5-FC at day 1. **B**: Semilogarithmic plot of individuals plasma concentration-time profile of 5-FC at day 1. **C**: Linear plot of individuals plasma concentration-time profile of 5-FC at day 7. **D**: Semilogarithmic plot of individuals plasma concentration-time profile of 5-FC at day 7

All pharmacokinetics parameters are presented in Table 1 and Additional file 1. Individual curves are presented in Fig. 1. As shown by Fig. 1, the elimination phase of the concentration curve was following a mono-exponential decrease.

**Table 1:** Non-compartmental pharmacokinetic parameters of oral 5-FC (100 mg/kg, twice daily) at days 1 and 7

Parameter	Unit	Day 1	Day 7
Elimination rate constant (λ_z_)	min^− 1^	0.0043 ± 0.0005	0.0039 ± 0.0002
Elimination half-life (t_1/2_)	min	163.1 ± 20.3	177.8 ± 11.0
Time to maximum plasma concentration (T_max_)	min	100 ± 17	120 ± 2
Maximum plasma concentration (C_max_)	µg/ml	84.5 ± 7.1	89.7 ± 12.3
Area under the concentration time curve (AUC_0 − inf_)	µg.min/ml	24 916 ± 2 076	30 174 ± 2 103*
Apparent volume of distribution at pseudo-equilibrium (Vz/F_obs)	ml/kg	936.9 ± 66.2	844.9 ± 25.7
Apparent clearance (Cl/F_obs)	ml/min/kg	4.0 ± 0.3	3.3 ± 0.2*

The asterisks indicate a significant difference (*p* < 0.05) between days 1 and 7 (paired t-test). Values are represented as means ± SD

The plasma concentration-time curve of 5-FC after the first administration showed a terminal elimination half-life (t_1/2_) of 163.1 ± 20.3 min which was not significantly different from the terminal half-life of 177.8 ± 11.0 min, calculated after 7 days of administration (Table 1). Based on the value of t_1/2_, we were able to compute the predicted accumulation ratio, which represents this accumulation of the drug between the first administration and the steady state situation. At steady-state, the concentration of the drug inside the body is in a dynamic equilibrium where the overall intake of the drug is equal to its elimination. Steady-state is generally obtained within 5 half-lives. Here, the accumulation ratio was between 3.3 and 7.2 %, depending on the value of the elimination rate constant (λ_z_) (as calculated by the formula $\frac{1}{1-{e}^{-{\uplambda }\text{z}\text{*}\text{T}\text{a}\text{u}}}$, Tau being the interval of administration). The maximal values of concentration (C_max_) at days 1 and 7 were not statistically significant (84.5 ± 7.1 and 89.7 ± 12.3 µg/ml, respectively) (Table 1).

Despite the low level of accumulation, apparent clearance was significantly decreased between days 1 and 7 (4.0 ± 0.3 and 3.3 ± 0.2 ml/min/kg, respectively, *p* = 0.009) (Table 1). Therefore, the area under the curve (AUC) showed a significant increase between days 1 and 7 (24 916 ± 2 076 and 30 174 ± 2 103 µg/ml.min, respectively, *p* = 0.004) (Table 1). No statistically significant differences were observed between the other pharmacokinetics parameters.

### Tolerance to 5-FC

**Fig. 2:**
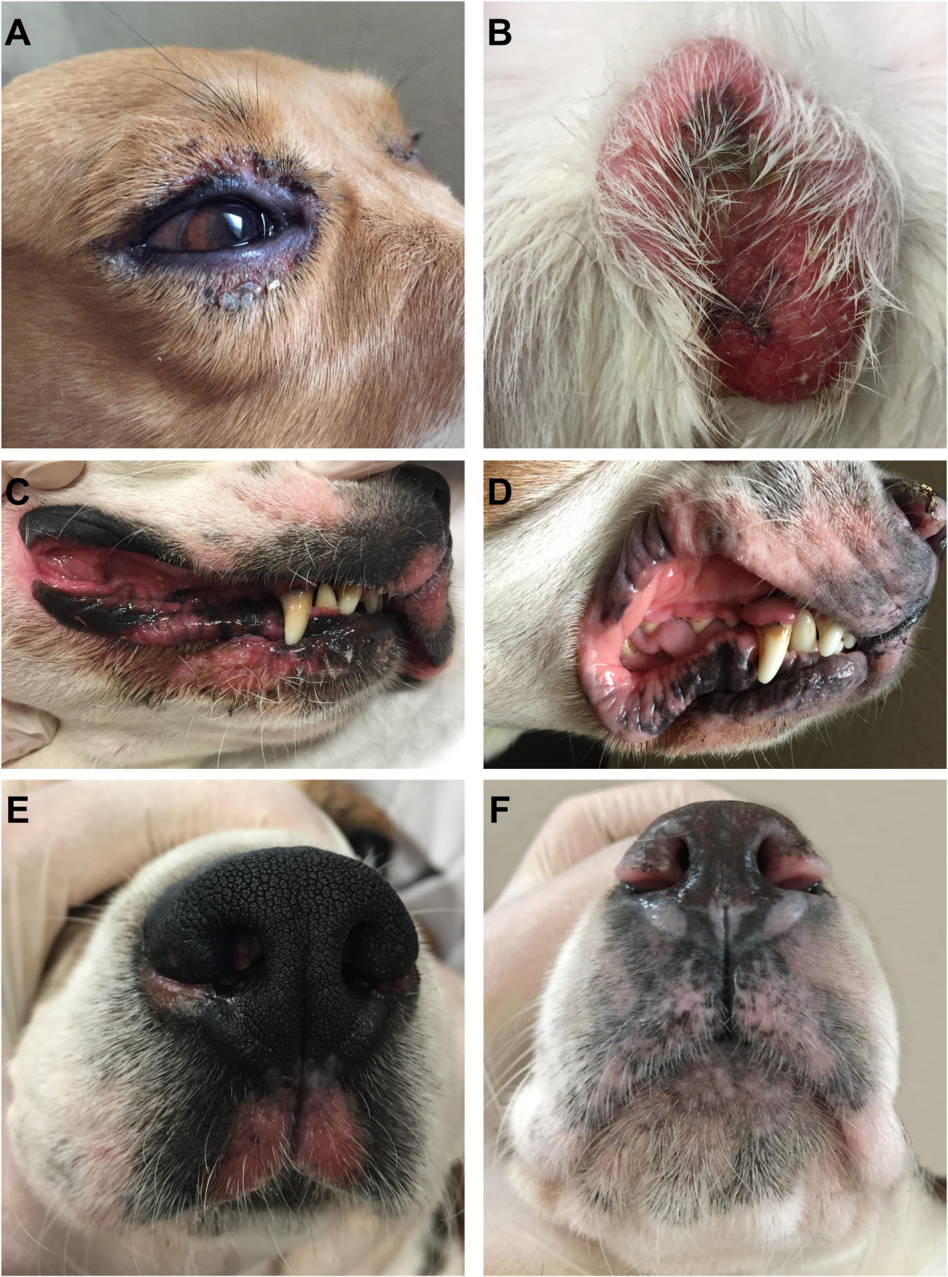
Cutaneous lesions in the dogs. **A**: Erythematous, ulcerated eyelid lesion at day 14. **B**: Erythematous, exudative lesion of the scrotum at day 14. **C**: Erythematous lips lesion at day 14. **D**: Depigmented lips lesion at day 24. **E**: Erythematous nasal planum lesion at day 14. **F**: Depigmented nasal planum lesion at day 28

All three dogs developed mucocutaneous or cutaneous lesions 10 (*n* = 2) to 13 days (*n* = 1) after 5-FC administration. Lesions were slightly erythematous (grade 1), slightly depigmented (grade 1), focally ulcerated (grade 1), exudative and mildly pruritic (grade 1) (Fig. 2). Fifteen to nineteen days after the onset of lesions a crust formation was observed. Lesions were relatively painless (grade 1). Lesions were localized on nasal planum (*n* = 3/3), lips (*n* = 3/3), eyelids (*n* = 3/3), skin (*n* = 1/3) and scrotum (*n* = 1/1) (Fig. 2). Sodium fusidate ointment (Fucidine 2 %, LEO Pharma, Voisins-le-Bretonneux, France) was applied twice daily until complete healing. Complete healing of lesions was noticed 15 days to 21 days after 5-FC discontinuation. No other clinical abnormality was noticed.

Hematological, biochemical and electrolytes analyses, performed at days 0, 7 and 14, did not reveal significant changes [see Additional files 2 and 3].

### Histological analyses

**Fig. 3:**
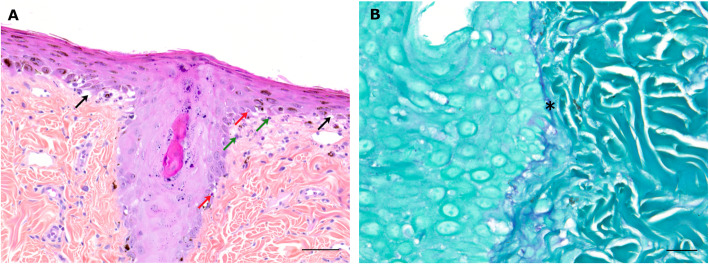
Histological microphotographs of cutaneous lesions. A: Interface dermatitis in nasal planum biopsy characterized by a moderate basal cell vacuolar degeneration (black arrows), a few apoptotic bodies in the basal layer (red arrows), and mild lymphocytic infiltrate (green arrows) at the dermal-epidermal junction. Hematoxylin-eosin-saffron staining. Scale bar: 100 μm. B: Nasal planum biopsy. The basement membrane (*) is thickened. Periodic acid-Schiff staining. Scale bar: 20 μm

Multifocal interface dermatitis, interspersed with areas of erosion and ulceration, was found in scrotal, labial and nasal biopsies from all dogs. The areas showing interface dermatitis were characterized by mild to moderate basal cell vacuolar degeneration, a few apoptotic bodies in the basal layer, and mild lymphocytic infiltrate at the dermal-epidermal junction (Fig. 3A). These lesions sometimes extended to the follicular infundibulum. The basement membrane was multifocally thickened (Fig. 3B). Pigmentary incontinence and/or mild to moderate acanthosis were often present. Ulcerated areas were covered with serocellular crusts and associated with moderate neutrophilic infiltrates in the superficial dermis. Areas of erosion displayed pustules and crusting and mild neutrophilic infiltrates in the superficial dermis with neutrophilic exocytosis across the epidermis.

## Discussion

This study describes pharmacokinetics and tolerance of repeated oral administration of 5-FC at 100 mg/kg in three healthy dogs, and has not been previously described in dogs. We conducted this study only on a very limited number of animals for obvious ethical reasons, as we looked at the toxic effects of 5-FC. The pharmacokinetics values and the statistics analysis then should be interpreted with extreme caution as the sample size was very low in our study.

Our data showed that 5-FC presents a mean terminal slope of 163.1 ± 20.3 min after the first administration, which is less than in human patients with normal renal function where half-life ranges between 3 and 8 h [32, 33]. Concomitant food intake has been demonstrated anecdotally to reduce the terminal half-life up to 2.6 h in human [34]. In our study, all animals were fasted 12 h before 5-FC administration and food intake is probably not interacting with the pharmacokinetics of 5-FC. The accumulation ratio computed from terminal half-life predicted a value of accumulation of 105 % as compared to baseline, which is consistent with the fact that we did not observed any significant differences between Cmax at day 1 and day 7. This confirms that steady state is obtained within 7 days. It is however unlikely but not impossible that accumulation occurred after the seventh day of administration and could lead to the cutaneous toxic manifestations that we observed. It is also important to note that in one of the animals, the exact C_max_ could have been missed as it seems to occur later than in other animals with his T_max_ at 120 min.

However, we saw an increase in AUC_0 − inf_ at a steady state compared to the first administration, with 30 174 ± 2 103 and 24 916 ± 2 076 µg.min/ml, respectively. It was related to a decrease in apparent clearance after 7 days, as compared to baseline values. Elimination of 5-FC is mostly by the kidney, as demonstrated in human patients, and alteration of clearance is generally related to kidney function impairment [34]. In our study, it is doubtful that kidney elimination of 5-FC was reduced during the seven days of treatment because the renal function parameters remained within the reference range [see Additional file 3]. Another explanation could be a decrease in the biotransformation of 5-FC (into other metabolites than 5-FU). A study investigating the metabolism differences of oral 5-FC administration at 100 mg/kg/day between humans and dogs demonstrated that dogs produced higher levels of metabolites (5 % of total dose) in urine as compared to humans (less than 0.5 %) [35]. Interestingly, in this study, no significant difference in metabolite rate was observed in dogs treated over 5 days with 5-FC, but we can hypothesize that a small decrease in metabolic rate could lead to AUC differences. We also cannot exclude a variation in oral bioavailability during the seven days of administration, as we did not specifically assess this parameter. Finally, as previously stated, the very low number of animals used here could led to interindividual variability and the differences in pharmacokinetics parameters that we observed should be interpreted carefully.

In humans, 5-FC toxicity is known to be related to C_max_ and the threshold value of 100 µg/ml is considered acceptable for avoiding any risk of thrombocytopenia or hepatotoxicity [7]. Hepatotoxicity accounts for 0 to 25 % of adverse effects in human medicine [1, 10, 36]. An increase in liver enzyme values is generally reversible with the reduction or the discontinuation of treatment [36, 37]. However, severe liver necrosis have been reported in patients treated for *Candida endocarditis* with 5-FC [12, 38]. In human medicine, severe bone marrow depression is reported with 5-FC treatment [8, 9, 36]. Cytopenia is observed in 60 % of patients when 5-FC blood concentration exceeds 100 µg/ml, while it occurs in 12 % of patients with 5-FC concentrations under 100 µg/ml [36]. In this study, we observed none of these side effects probably because C_max_ was consistently below 100 µg/ml in all dogs. Only one dog experienced transient diarrhea with an increase of up to 2 stools per day and a decrease in consistency over baseline (grade 1). In human medicine, gastrointestinal side effects occurred in 6 % of patients treated with 5-FC [6]. Although these side effects are usually not severe, cases of ulcerative colitis and bowel perforation have been reported [6, 39–44].

Despite the absence of liver or bone marrow toxicity, we did see cutaneous toxicity. In our study, mucocutaneous or cutaneous lesions were observed 10 to 13 days after the first administration of 5-FC for a cumulative dose ranging from 2.0 to 2.6 g/kg. Mucocutaneous or cutaneous lesions have been reported in dogs treated for cryptococcosis and aspergillosis with a median dose of 5-FC of 150 mg/kg/day (range: 105–188 mg/kg) divided in three equal doses with amphotericin B (0.5 to 0.8 mg/kg by subcutaneous or slow intravenous infusion twice or thrice weekly) [3]. The median time before developing lesions was 20 days (range: 13 to 41 days) [3]. Development of drug eruptions seemed more rapid in dogs concurrently receiving thiazoles like amphotericin B than without, respectively 18.5 days and 38 days [3]. It suggests that interactions with cytochrome P450 could be responsible for the higher risk of toxicity.

As described by Malik et *al*., and Panciera and Bevier, lesions observed in our study were pruritic, depigmented, ulcerated and exudative and evolved into crust lesions [2, 3]. Lesions were localized on the lips, nostril, philtrum, eyelids, scrotum and nipples. Similar localizations have been reported by Malik et *al.*, with scrotum (*n* = 4/6), nasal planum (*n* = 6/7), lips (*n* = 3/7), vulva (*n* = 1/4), external ear canal (*n* = 1/7) and skin (*n* = 2/7) involvement [3]. In our study, histological analysis of mucocutaneous and cutaneous lesions revealed interface dermatitis with basal cell vacuolar degeneration, a few apoptotic bodies in the basal layer, mild lymphocytic infiltrate at the dermal-epidermal junction, increased thickness of the basement membrane, with multifocal erosion and ulceration. These histologic findings mimic the histologic features of cutaneous lupus erythematosus, especially the mucocutaneous variant of cutaneous lupus erythematosus [45, 46]. Combined with the history and the clinical data, the histological findings are consistent with a lupoid drug reaction [45, 47]. Other reports of 5-FC induced cutaneous lesions in dogs documented toxic epidermal necrolysis or fixed drug eruption [2, 48]. Histologic features of toxic epidermal necrolysis include diffuse devitalization of the epidermis with mild dermal inflammation [45]. In our study, we did not see preexisting diffuse epidermal devitalization, characterized as the degree of epidermal and follicular compromise, at the ulcers’ margins. Fixed drug eruption is characterized by lesions at the same body site each time the patient is challenged with the drug. The histological lesions are similar to those of erythema multiforme which is characterized by an interface dermatitis with keratinocyte apoptosis and satellitosis [45, 48]. In our study, we did find keratinocyte apoptosis, but it was not a prominent feature; satellitosis was seldom identified. In human patients, lupoid drug reactions have been recognized in association with 118 drugs [49]. It has not been associated with 5-FC medication but with intravenous administration of 5-FU [50]. However, in our study blood concentration of 5-FU was below the limit of detection for all dogs.

The mechanism of toxicity of 5-FC is not fully understood [12]. Dose dependent toxicity is mainly suspected of hepatic and bone marrow toxicity, although not all reports support this theory. Conversion of 5-FC to certain metabolites, like 5-FU, is one of the suspected mechanisms. Patients treated with 5-FC have been shown to have detectable amounts of 5-FU in serum leading to bone marrow depression and gastrointestinal adverse events [13]. In healthy volunteers, serum 5-FU concentrations ranged from 10 to 400 ng/ml during six hours after oral administration of 2 g of 5-FC [13]. *In vitro* induction of enzymes responsible for deamination of 5-FC to 5-FU has been assessed in the intestinal microflora after chronic exposure to 5-FC (50 mg/day) [14]. A relationship between the gut flora level, particularly the gram-negative enterobacilli, and the conversion of 5-FC to 5-FU has been identified in two patients [15]. Despite chronic administration of 5-FC, blood 5-FU concentrations were below the limit of detection in our study.

Targeted tumor chemotherapy is a promising approach to concentrate drugs within a tumor while minimizing the toxicity of chemotherapy. Oncolytic viruses can efficiently target and express transgenes within a tumor and cause drug accumulation in order to kill tumor cells. TG6002 is a Copenhagen strain of vaccinia virus with deletions of thymidine kinase (*TK; J2R*) and ribonucleotide reductase (*RR; I4L*) genes [30]. TG6002 is armed with the therapeutic gene *FCU1*, which encodes a bifunctional chimeric protein that catalyzes the conversion of 5-FC into the toxic metabolites 5-FU and 5-FUMP [51]. TG6002 has been shown to replicate and exert oncolytic potency in canine cell lines and canine xenograft model [52]. In murine xenograft models of hepatocarcinoma and colorectal cancer treated with intravenous administration of TG6002 and oral 5-FC, a significant reduction of tumor size and an intratumoral production of 5-FU were reported [30]. The lytic properties of TG6002 and the 5-FU synthesis were also assessed on canine mammary tumor explants. *In vitro* infection of canine mammary carcinoma biopsies with TG6002 led to tumor necrosis and the conversion of 5-FC into 5-FU [52]. From a clinical trial perspective, a safety study has established the innocuity of multiple intramuscular injections of TG6002 with short term administration of 5-FC in healthy dogs [31]. These promising results have supported the use of TG6002 in combination with 5-FC in a clinical trial in both dogs an humans (NCT03724071, NCT04194034) [53, 54]. For its use in canine targeted chemotherapy, an adaptation of the protocol of administration of 5-FC might be considered in order to limit 5-FC cutaneous toxicity. As tumors are well vascularized, allowing for a larger tumoral 5-FC input, either a reduction of 5-FC dosage or an increase in administration interval could be considered.

The main limitations of this study relate to the small number of dogs involved and the statistics need to be interpreted with extreme caution. Another limitation of our study is also that we only assessed the 5-FU concentration and did not looked for any potential other metabolites for 5-FC.

## Conclusions

Cutaneous drug lupoid reaction was the main toxicity of continuous oral 5-FC administration at 100 mg/kg twice daily for 10 days. No other adverse events were noticed. Toxicity might not be secondary to 5-FU production but more likely be induced by 5-FC or other 5-FC metabolites. Based on those data, an adjustment of 5-FC administration will be needed for targeted chemotherapy.

## Methods

### Laboratory dogs

Two female and one male adult healthy Beagle dogs (Harlan Laboratories, Gannat, France) were used. The weight of the dog was between 11.0 and 16.9 kg. All dogs were acclimatized for seven days before starting the experiment and were under the care of a licensed veterinarian. The dogs were housed individually in stainlees-steel bar boxes with a resin soil substrate and a shaved softwood litter. The room temperature was 19 °C (+/- 2 °C) with a humidity greater than 35 %, and the day/night cycle was 12:12 h. Dogs were fed twice daily with a certified commercial canine diet and given potable water *ad libitum*. Dogs were not euthanized at the end of the study. All the dogs that participated were rehomed as companion animals one month after the end of the study.

### 5-FC

5-FC (Toronto Research Chemicals, North York, ON, Canada) was compounded into 500 mg or 100 mg tablets by the pharmacy of the Veterinary University Hospital of Alfort. 5-FC was stored at a temperature under 25 °C and protected from moisture. 5-FC was administered in a dedicated room of the laboratory animal facility and outside the room housing the dogs.

### Study design

 This study was conducted in accordance with European legislation and French regulations on the protection of animals used for scientific purposes (Directive 2010/63/EU, 2010; Code rural, 2018; Décret no. 2013 − 118, 2013) and complied to the recommendations of the “Charte nationale portant sur l’éthique en expérimentation animale” established by the “Comité National de Réflexion Ethique sur l’Expérimentation Animale” (CNREEA—Ministère de l’Enseignement Supérieur, de la Recherche et de l’Innovation—Ministère de l’Agriculture et de l’Alimentation). The study protocol (N° 19–047) was approved by the Anses/EnvA/UPEC ethical committee (N° 16). The study was carried out in compliance with the ARRIVE guidelines.

The dogs received 100 mg/kg of 5-FC twice a day, by oral route. 5-FC was administered until the development of adverse events. Dogs were fasted 12 h before 5-FC administration and fed one hour after. The dose chosen in this study was similar to those used in veterinary literature [24].

Dogs were evaluated once-a-day by a physical examination during the study. On days 0, 7 and 14 complete blood counts were performed using a Sysmex XT automated hematology analyzer (Sysmex France, Villepinte, France); biochemistry analyses were performed using a Vitalab Selectra XL biochemistry analyzer (ELITech, Puteaux, France); and electrolyte analyses were performed on days 0, 7 and 14 using a Nova CRT 8 electrolyte analyzer (Nova Biomedical, Les Ulis, France).

Serum 5-FC and 5-FU assays were performed on days 1 and 7. Blood samples were collected 15, 30, 45, 60, 90 and 120 min after 5-FC administration, and 6, 8 and 11 h after administration. Samples were centrifuged at 5 000 revolutions per minute at 4 °C for 5 min and stored at -80 °C until analysis.

### Adverse events

Assessment of adverse events was performed according to the Veterinary Cooperative Oncology Group Common Terminology Criteria for Adverse Events guidelines [55]. Throughout the study, adverse events were monitored by daily physical examination, complete blood count, biochemistry, and electrolyte analyses.

### 5-FC and 5-FU serum concentration

Quantification of 5-FC and 5-FU was performed on serum by high-performance liquid chromatography (HP Agilent 1100 series, Agilent Technologies, Santa Cruz, CA, USA). Plasma samples were quenched with acetonitrile, 50 % (v/v). Supernatant was reconstituted in 60 µl of water. Separation of 5-FC and 5-FU was accomplished under isocratic conditions using a RP-C18 column (Supelco supelcosil LC-18-S; 5 μm packing; 4.6 × 250 mm; Supelco, Sigma-Aldrich Chimie Sarl, Lyons, France) and a guard cartridge (ChromGuard RP 10 × 3mm, Agilent Technologies, Santa Cruz, CA, USA) with a mobile phase of orthophosphoric acid (50 mM, pH 2.1) for 16 min and an elution flow of 0.8 mL/min. Spectrophotometric diode array detection was used at 260 nm and 280 nm. Sample concentrations of 5-FC and 5-FU were calculated respectively based on a standard curve of 5-FC and 5-FU.

### Pharmacokinetics analysis

Serum samples of 5-FC obtained on days 1 and 7, were independently analyzed by non-compartimental analysis using PKanalix software. Maximum plasma concentration (C_max_), time to maximum plasma concentration (T_max_), and elimination rate constant (λ_z_) of the log-linear slop for concentration versus time regression line was computed. Based on these parameters, the area under the concentration-time curve (AUC) was calculated using a linear up-log down trapezoidal rule from time 0 to the last time of sample collection and extrapolated to infinity by adding AUC trapezoid value of C_last_/λ_z_, C_last_ being the last quantifiable 5-FC plasma concentration. Similarly, area under first moment (AUMC) was calculated. Elimination half-life (t_1/2_) was calculated using ln(2)/ λ_z_. Apparent clearance (CL/F) was calculated as Dose/AUC and apparent volume of distribution at pseudo-equilibrium (V_z_/F) as Cl/F/ λ_z_. Mean Residency Time was calculated as AUMC/AUC. Each parameter was expressed as mean ± SD. Parameters were compared for days 1 and 7 by paired t-test. Statistical significance was set at *p* < 0.05.

### Histological analyses

Skin biopsies were performed under general anesthesia. Anesthesia was performed using an initial intravenous administration of 0.2 mg/kg of butorphanol (Torbugesic, Zoetis, Malakoff, France), 3 mg/kg of ketamine (Ketamine 1000, Virbac, Carros, France) and 15 µg/kg of medetomidine (Domitor, Orion Corporation, Espoo, Finland). Analgesia was performed by subcutaneous injection of lidocaine (Lidor, Axience, Pantin, France) mixed with sodium bicarbonate in a 1:1 ratio into the skin surrounding the lesion. Total dose of lidocaine did not exceed 5 mg/kg. Biopsies of skin lesions were performed using a 6 mm skin punch biopsy (Skin biopsy punch 273,690, Kruuse, Langeskov, Denmark). A single cruciate suture using non-absorbable monofilament (Ethilon 3 − 0, Ethicon, Somerville, New Jersey, USA) was used to suture biopsy sites. Samples were fixed in 10 % buffered formalin for 48 h and then embedded in paraffin, routinely processed, sliced at 4 μm, stained with hematoxylin-eosin-saffron and Periodic acid-Schiff and examined by light microscopy. Slides were evaluated by two European College of Veterinary Pathologists diplomates (EL and FD).

## Supplementary Information

**Additional file 1.** Individual values of the pharmacokinetics parameters computed for each dog at days 1 and 7.

**Additional file 2.** Complete blood count of dogs treated with 5-FC (100mg/kg, twice daily) at days 0, 1 and 7. No significant anomalies were noted. Bold numbers refer to values outside the reference interval.

**Additional file 3.** Biochemical analyses of dogs treated with 5-FC (100mg/kg, twice daily) at days 0, 1 and 7. No significant anomalies were noted. Bold numbers refer to values outside the reference interval.

## Data Availability

The datasets used and/or analysed during the current study are available from the corresponding author on reasonable request.

## Abbreviations

5-FC: 5-fluorocytosine
5-FU: 5-fluorouracil
5-FUMP: 5-fluorouridine monophosphate
5-FdUMP: 5-fluorodeoxyuridine monophosphate
AUC: Area under the curve
C_max_: Maximal values of concentration
t_1/2_: Elimination half-life
λ_z_: Elimination rate constant

## References

[1] Bennett JE, Flucytosine (1977). Ann Intern Med.

[2] Panciera DL, Bevier D (1987). Management of cryptococcosis and toxic epidermal necrolysis in a dog. J Am Vet Med Assoc.

[3] Malik R, Medeiros C, Wigney DI, Love DN (1996). Suspected drug eruption in seven dogs during administration of flucytosine. Aust Vet J.

[4] Malik R, Craig AJ, Wigney DI, Martin P, Love DN (1996). Combination chemotherapy of canine and feline cryptococcosis using subcutaneously administered amphotericin B. Aust Vet J.

[5] Green NK, Herbert CW, Hale SJ, Hale AB, Mautner V, Harkins R (2004). Extended plasma circulation time and decreased toxicity of polymer-coated adenovirus. Gene Ther.

[6] Benson JM, Nahata MC (1988). Clinical use of systemic antifungal agents. Clin Pharm..

[7] Vermes A, van Der Sijs H, Guchelaar HJ (2000). Flucytosine: correlation between toxicity and pharmacokinetic parameters. Chemotherapy.

[8] Meyer R, Axelrod JL (1974). Fatal aplastic anemia resulting from flucytosine. JAMA.

[9] Kauffman CA, Frame PT (1977). Bone marrow toxicity associated with 5-fluorocytosine therapy. Antimicrob Agents Chemother.

[10] Bennett JE, Dismukes WE, Duma RJ, Medoff G, Sande MA, Gallis H (1979). A comparison of amphotericin B alone and combined with flucytosine in the treatment of cryptoccal meningitis. N Engl J Med.

[11] Utz JP, Garriques IL, Sande MA, Warner JF, Mandell GL, McGehee RF (1975). Therapy of cryptococcosis with a combination of flucytosine and amphotericin B. J Infect Dis.

[12] Vermes A (2000). Flucytosine: a review of its pharmacology, clinical indications, pharmacokinetics, toxicity and drug interactions. J Antimicrob Chemother.

[13] Diasio RB, Lakings DE, Bennett JE (1978). Evidence for conversion of 5-fluorocytosine to 5-fluorouracil in humans: possible factor in 5-fluorocytosine clinical toxicity. Antimicrob Agents Chemother.

[14] Harris BE, Manning BW, Federle TW, Diasio RB (1986). Conversion of 5-fluorocytosine to 5-fluorouracil by human intestinal microflora. Antimicrob Agents Chemother.

[15] Malet-Martino M-C, Martino R, de Forni M, Andremont A, Hartmann O, Armand JP (1991). Flucytosine conversion to fluorouracil in humans: does a correlation with gut flora status exist? A report of two cases using fluorine-19 magnetic resonance spectroscopy. Infection.

[16] Kufe DW, Major PP (1981). 5-Fluorouracil incorporation into human breast carcinoma RNA correlates with cytotoxicity. J Biol Chem.

[17] Kufe DW, Major PP, Egan EM, Loh E (1981). 5-Fluoro-2’-deoxyuridine incorporation in L1210 DNA. J Biol Chem.

[18] Tanaka M, Yoshida S, Saneyoshi M, Yamaguchi T (1981). Utilization of 5-fluoro-2’-deoxyuridine triphosphate and 5-fluoro-2’-deoxycytidine triphosphate in DNA synthesis by DNA polymerases alpha and beta from calf thymus. Cancer Res.

[19] Santi DV, McHenry CS, Sommer H (1974). Mechanism of interaction of thymidylate synthetase with 5-fluorodeoxyuridylate. Biochemistry.

[20] Menard K, Flesner BK, Glahn A, Boudreaux B, Bryan JN (2018). Concurrent 5-fluorouracil and carboplatin for the treatment of canine carcinomas. Vet Comp Oncol.

[21] Karayannopoulou M, Kaldrymidou E, Constantinidis TC, Dessiris A (2001). Adjuvant post-operative chemotherapy in bitches with mammary cancer. J Vet Med A Physiol Pathol Clin Med.

[22] Stanclift RM, Gilson SD. Use of cisplatin, 5-fluorouracil, and second-look laparotomy for the management of gastrointestinal adenocarcinoma in three dogs. J Am Vet Med Assoc. 2004;225:1412–7, 1393. 10.2460/javma.2004.225.1412 15552318

[23] Henry CJ, Brewer WG, Tyler JW, Brawner WR, Henderson RA, Hankes GH (1998). Survival in dogs with nasal adenocarcinoma: 64 cases (1981–1995). J Vet Intern Med.

[24] Plumb DC. Plumb’s veterinary drug handbook. 9th edition. Stockholm, Wisconsin: Pharma Vet Inc; 2018.

[25] Børresen B, Hansen AE, Kjaer A, Andresen TL, Kristensen AT (2018). Liposome-encapsulated chemotherapy: Current evidence for its use in companion animals. Vet Comp Oncol.

[26] Oerlemans C, Bult W, Bos M, Storm G, Nijsen JFW, Hennink WE (2010). Polymeric Micelles in Anticancer Therapy: Targeting, Imaging and Triggered Release. Pharm Res.

[27] Tan A, Yildirimer L, Rajadas J, De La Peña H, Pastorin G, Seifalian A (2011). Quantum dots and carbon nanotubes in oncology: a review on emerging theranostic applications in nanomedicine. Nanomed.

[28] Zabielska-Koczywąs K, Lechowski R (2017). The use of liposomes and nanoparticles as drug delivery systems to improve cancer treatment in dogs and cats. Molecules.

[29] Alyassin Y, Sayed EG, Mehta P, Ruparelia K, Arshad MS, Rasekh M (2020). Application of mesoporous silica nanoparticles as drug delivery carriers for chemotherapeutic agents. Drug Discov Today.

[30] Foloppe J, Kempf J, Futin N, Kintz J, Cordier P, Pichon C (2019). The enhanced tumor specificity of TG6002, an armed oncolytic vaccinia virus deleted in two genes involved in nucleotide metabolism. Mol Ther Oncolytics.

[31] Béguin J, Nourtier V, Gantzer M, Cochin S, Foloppe J, Balloul J-M (2020). Safety studies and viral shedding of intramuscular administration of oncolytic vaccinia virus TG6002 in healthy beagle dogs. BMC Vet Res.

[32] Block ER (1974). Flucytosine and amphotericin B: hemodialysis effects on the plasma concentration and clearance: studies in man. Ann Intern Med.

[33] Pai MP, Bruce H, Felton LA (2010). Clinical pharmacokinetics of oral controlled-release 5-fluorocytosine. Antimicrob Agents Chemother.

[34] Cutler RE, Blair AD, Kelly MR (1978). Flucytosine kinetics in subjects with normal and impaired renal function. Clin Pharmacol Ther.

[35] Polak A, Eschenhof E, Fernex M, Scholer HJ (1976). Metabolic studies with 5-fluorocytosine-6-^14^ C in mouse, rat, rabbit, dog and man. Chemotherapy.

[36] Stamm AM, Diasio RB, Dismukes WE, Shadomy S, Cloud GA, Bowles CA (1987). Toxicity of amphotericin B plus flucytosine in 194 patients with cryptococcal meningitis. Am J Med.

[37] Francis P, Walsh TJ (1992). Evolving role of flucytosine in immunocompromised patients: new insights into safety, pharmacokinetics, and antifungal therapy. Clin Infect Dis.

[38] Spernovasilis N, Kofteridis D (2018). Pre-existing liver disease and toxicity of antifungals. J Fungi.

[39] Patel R. Antifungal Agents. Part I. Amphotericin B preparations and flucytosine. Mayo Clin Proc. 1998;73:1205–25. 10.4065/73.12.1205 9868423

[40] Sohail MA, Ikram U. Flucytosine-induced colitis. BMJ Case Rep. 2014;2014:1–3. 10.1136/bcr-2013-203381 PMC4009913 24777084

[41] White CA, Traube J (1982). Ulcerating enteritis associated with flucytosine therapy. Gastroenterology.

[42] Fond B, Bentata-Pessayre M, Krivitzky A, Callard P, Dupont B, Delzant G (1983). Iatrogenic colitis during flucytosine treatment for neuromeningeal cryptococcosis. Sem Hopitaux Organe Fonde Par Assoc Enseign Med Hopitaux Paris.

[43] Fortson WC, Tedesco FJ (1984). Drug-induced colitis: a review. Am J Gastroenterol.

[44] Cappell MS, Simon T (1993). Colonic toxicity of administered medications and chemicals. Am J Gastroenterol.

[45] Gross TL, Ihrke PJ, Walder EJ, Affolter VK (2005). Skin diseases of the dog and cat.

[46] Olivry T, Rossi MA, Banovic F, Linder KE (2015). Mucocutaneous lupus erythematosus in dogs (21 cases). Vet Dermatol.

[47] Voie KL, Campbell KL, Lavergne SN (2012). Drug hypersensitivity reactions targeting the skin in dogs and cats. J Vet Intern Med.

[48] Mason KV (1993). Blistering drug eruptions in animals. Clin Dermatol.

[49] Arnaud L, Mertz P, Gavand P-E, Martin T, Chasset F, Tebacher-Alt M (2019). Drug-induced systemic lupus: revisiting the ever-changing spectrum of the disease using the WHO pharmacovigilance database. Ann Rheum Dis.

[50] Almagro BM, Steyls MC, Navarro NL, Domínguez EG, Acosta EH, Pérez MAG (2011). Occurrence of subacute cutaneous lupus erythematosus after treatment with systemic fluorouracil. J Clin Oncol Off J Am Soc Clin Oncol.

[51] Erbs P, Regulier E, Kintz J, Leroy P, Poitevin Y, Exinger F (2000). In vivo cancer gene therapy by adenovirus-mediated transfer of a bifunctional yeast cytosine deaminase/uracil phosphoribosyltransferase fusion gene. Cancer Res.

[52] Béguin J, Foloppe J, Maurey C, Laloy E, Hortelano J, Nourtier V (2020). Preclinical evaluation of the oncolytic vaccinia virus TG6002 by translational research on canine breast cancer. Mol Ther - Oncolytics.

[53] Clinical Trials.gov. National Library of Medicine (US). Identifier NCT03724071, study of TG6002 (VV TK-RR-FCU1) in combination with 5-FC in patients with advanced gastro-intestinal tumors; 2018. Available from: https://clinicaltrials.gov/ct2/show/NCT03724071.

[54] Clinical Trials.gov. National Library of Medicine (US). Identifier NCT04194034, study of intrahepatic arterial infusion of TG6002 in combination with 5-FC in patients with metastatic colorectal cancer; 2019. Available from: https://clinicaltrials.gov/ct2/show/NCT04194034.

[55] Veterinary cooperative oncology group (2016). - common terminology criteria for adverse events (VCOG-CTCAE) following chemotherapy or biological antineoplastic therapy in dogs and cats v1.1. Vet Comp Oncol.

