# Supplementary material for: Pharmacokinetics and tolerance of repeated oral administration of 5-fluorocytosine in healthy dogs
Source: BMC Vet Res. 2021 Jun 21;17:220. doi: 10.1186/s12917-021-02927-5 (PMC8218522; doi:10.1186/s12917-021-02927-5)
Supplement: Supplementary file 1 — Additional file 1. Individual values of the pharmacokinetics parameters computed for each dog at days 1 and 7. [file 12917_2021_2927_MOESM1_ESM.docx]

| **Parameter** | **Unit** | **Dog1** | **Dog 2** | **Dog 3** |
| --- | --- | --- | --- | --- |
| Elimination rate constant (λ_z_) | min^-1^ | 0.00437 | 0.00374 | 0.00477 |
| Elimination half-life (t_1/2_) | min | 158.69 | 185.27 | 145.30 |
| Time to maximum plasma concentration (T_max_) | min | 90 | 90 | 120 |
| Maximum plasma concentration (C_max_) | μg/ml | 92.27 | 78.33 | 82.9 |
| Area under the concentration time curve (AUC_0-inf_) | µg.min/ml | 25632 | 26540 | 22576 |
| Apparent volume of distribution at pseudo-equilibrium (Vz/F_obs) | ml/kg | 893 | 1013 | 904 |
| Apparent clearance (Cl/F_obs) | ml/min/kg | 3.90 | 3.79 | 4.31 |

**Individual values of the pharmacokinetics parameters at day 1**

| **Parameter** | **Unit** | **Dog1** | **Dog 2** | **Dog 3** |
| --- | --- | --- | --- | --- |
| Elimination rate constant (λ_z_) | min^-1^ | 0.00387 | 0.00369 | 0.00417 |
| Elimination half-life (t_1/2_) | min | 179.25 | 187.99 | 166.15 |
| Time to maximum plasma concentration (T_max_) | min | 122 | 119 | 120 |
| Maximum plasma concentration (C_max_) | μg/ml | 93.79 | 99.44 | 75.88 |
| Area under the concentration time curve (AUC_0-inf_) | µg.min/ml | 31492 | 31283 | 27749 |
| Apparent volume of distribution at pseudo-equilibrium (Vz/F_obs) | ml/kg | 821 | 872 | 841 |
| Apparent clearance (Cl/F_obs) | ml/min/kg | 3.18 | 3.22 | 3.51 |

**Individual values of the pharmacokinetics parameters at day 7**
